# 1,4-Dihydro­quinoxaline-2,3-dione–5-nitro­isophthalic acid–water (1/1/1)

**DOI:** 10.1107/S1600536811020873

**Published:** 2011-06-04

**Authors:** Ming-Feng Wang

**Affiliations:** a Department of Chemistry and Chemical Engineering, Jining University, 273155 Qufu, Shandong, People’s Republic of China

## Abstract

The asymmetric unit of the title compound, C_8_H_6_N_2_O_2_·C_8_H_5_NO_6_·H_2_O, contains mol­ecules of 1,4-dihydro­quinoxaline-2,3-dione, 5-nitro­isophthalic acid and a solvent water. In the crystal structure, mol­ecules are linked into a three-dimensional network by inter­molecular N—H⋯O and O—H⋯O hydrogen bonds.

## Related literature

For applications of piperazine and its derivatives, see: Jian & Zhao (2004 ▶); Oxtoby *et al.* (2005 ▶). For uses of 5-nitro­isophthalate and its derivatives, see: He *et al.* (2004 ▶); Wang *et al.* (2009 ▶); Xu *et al.* (2011 ▶). For bond-length data, see: Allen *et al.* (1987 ▶).
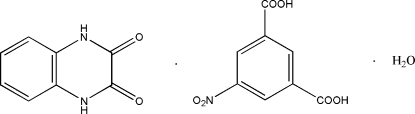


## Experimental

### 

#### Crystal data

C_8_H_6_N_2_O_2_·C_8_H_5_NO_6_·H_2_O
*M* *_r_* = 391.29
Triclinic, 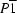

*a* = 7.245 (2) Å
*b* = 8.686 (3) Å
*c* = 13.142 (4) Å
α = 93.938 (4)°
β = 95.619 (4)°
γ = 95.793 (4)°
*V* = 816.3 (4) Å^3^
*Z* = 2
Mo *K*α radiation
μ = 0.13 mm^−1^
*T* = 295 K
0.20 × 0.16 × 0.10 mm

#### Data collection

Bruker APEXII CCD diffractometer
Absorption correction: multi-scan (*SADABS*; Bruker, 2005 ▶) *T*_min_ = 0.974, *T*_max_ = 0.987
4482 measured reflections
2857 independent reflections
2441 reflections with *I* > 2σ(*I*)
*R*_int_ = 0.017

#### Refinement

*R*[*F* ^2^ > 2σ(*F* ^2^)] = 0.033
*wR*(*F* ^2^) = 0.095
*S* = 1.03
2857 reflections
254 parameters
H-atom parameters constrained
Δρ_max_ = 0.18 e Å^−3^
Δρ_min_ = −0.19 e Å^−3^

### 

Data collection: *APEX2* (Bruker, 2005 ▶); cell refinement: *SAINT* (Bruker, 2005 ▶) and *APEX2*; data reduction: *SAINT*; program(s) used to solve structure: *SHELXTL* (Sheldrick, 2008 ▶); program(s) used to refine structure: *SHELXTL*; molecular graphics: *SHELXTL*; software used to prepare material for publication: *SHELXTL*.

## Supplementary Material

Crystal structure: contains datablock(s) global, I. DOI: 10.1107/S1600536811020873/cv5099sup1.cif

Structure factors: contains datablock(s) I. DOI: 10.1107/S1600536811020873/cv5099Isup2.hkl

Supplementary material file. DOI: 10.1107/S1600536811020873/cv5099Isup3.cml

Additional supplementary materials:  crystallographic information; 3D view; checkCIF report

## Figures and Tables

**Table 1:** Hydrogen-bond geometry (Å, °)

*D*—H⋯*A*	*D*—H	H⋯*A*	*D*⋯*A*	*D*—H⋯*A*
N2—H2⋯O6^i^	0.86	2.40	2.9632 (18)	123
N2—H2⋯O9^ii^	0.86	2.33	3.0071 (17)	136
N1—H1⋯O1^iii^	0.86	2.02	2.8723 (17)	173
O9—H9*B*⋯O2^iv^	0.86	1.89	2.7456 (15)	171
O8—H8⋯O3^v^	0.82	1.86	2.6381 (17)	159
O9—H9*A*⋯O1	0.86	1.97	2.8220 (15)	168
O4—H4*A*⋯O9	0.82	1.78	2.5962 (15)	173

Symmetry codes: (i) 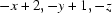; (ii) 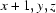; (iii) 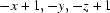; (iv) 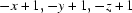; (v) 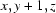.

**Fig. 1.**
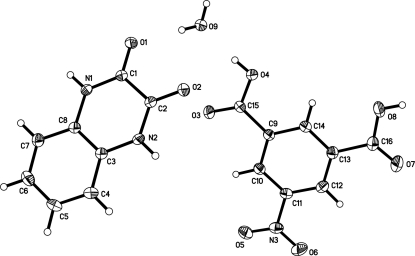
The content of asymmetric unit of (I) showing the atomic labeling and 30% probability displacement ellipsoids.

## supplementary crystallographic information

### Comment

Piperazine and its derivatives have attracted a great interest due to their use as curatorial intermediate, bacteriophage and insectifuge (Jian & Zhao, 2004; Oxtoby *et al.*, 2005). Coordination polymers of 5-nitroisophthalate and its derivatives have attracted interest because of their potential applications and intriguing architectures with new topologies (He *et al.*, 2004; Wang *et al.*, 2009; Xu *et al.*, 2011). In this paper, we present the title compound (I).

In (I) (Fig. 1), the bond lengths and angles are normal (Allen *et al.*, 1987). The asymmetric unit contains one molecule of 1,4-dihydro-2,3-quinoxalinedione, one molecule of 5-nitro-isophthalic acid and one crystalline water molecule.

The crystal packing is stabilized by intermolecular N—H···O and O—H···O hydrogen bonds (Table 1), which link the molecules into three-dimensional network.

### Experimental

A water solution (50 ml) of 1,4-Dihydro-2,3-quinoxalinedione (0.25 mmol) and 5-nitro-isophthalic acid (0.25 mmol) was heated at 333 K for 3 h. Then the mixture was cooled to room temperature. After two weeks orange crystals suitable for X-ray diffraction study were obtained.

### Refinement

All H atoms were positioned geometrically and refined using a riding model approximation with C—H = 0.93 Å, N—H = 0.86 Å, O_carbonyl_—H = 0.82 Å and O_water_—H = 0.86 Å and with *U*_iso_(H) = 1.2*U*_eq_(C,N) and *U*_iso_(H) = 1.5*U*_eq_(O), respectively.

### Crystal data

**Table:**

C_8_H_6_N_2_O_2_·C_8_H_5_NO_6_·H_2_O	*Z* = 2
*M**_r_* = 391.29	*F*(000) = 404
Triclinic, *P*1	*D*_x_ = 1.592 Mg m^−^^3^
Hall symbol: -P 1	Mo *K*α radiation, λ = 0.71073 Å
*a* = 7.245 (2) Å	Cell parameters from 2442 reflections
*b* = 8.686 (3) Å	θ = 2.4–28.2°
*c* = 13.142 (4) Å	µ = 0.13 mm^−^^1^
α = 93.938 (4)°	*T* = 295 K
β = 95.619 (4)°	Block, orange
γ = 95.793 (4)°	0.20 × 0.16 × 0.10 mm
*V* = 816.3 (4) Å^3^	

### Data collection

**Table:**

Bruker APEXII CCD diffractometer	2857 independent reflections
Radiation source: fine-focus sealed tube	2441 reflections with *I* > 2σ(*I*)
graphite	*R*_int_ = 0.017
φ and ω scans	θ_max_ = 25.1°, θ_min_ = 1.6°
Absorption correction: multi-scan (*SADABS*; Bruker, 2005)	*h* = −8→8
*T*_min_ = 0.974, *T*_max_ = 0.987	*k* = −9→10
4482 measured reflections	*l* = −15→15

### Refinement

**Table:**

Refinement on *F*^2^	Secondary atom site location: difference Fourier map
Least-squares matrix: full	Hydrogen site location: inferred from neighbouring sites
*R*[*F*^2^ > 2σ(*F*^2^)] = 0.033	H-atom parameters constrained
*wR*(*F*^2^) = 0.095	*w* = 1/[σ^2^(*F*_o_^2^) + (0.0491*P*)^2^ + 0.199*P*] where *P* = (*F*_o_^2^ + 2*F*_c_^2^)/3
*S* = 1.03	(Δ/σ)_max_ < 0.001
2857 reflections	Δρ_max_ = 0.18 e Å^−^^3^
254 parameters	Δρ_min_ = −0.19 e Å^−^^3^
0 restraints	Extinction correction: *SHELXTL* (Sheldrick, 2008), Fc^*^=kFc[1+0.001xFc^2^λ^3^/sin(2θ)]^-1/4^
Primary atom site location: structure-invariant direct methods	Extinction coefficient: 0.040 (3)

### Special details

**Table:**

Geometry. All e.s.d.'s (except the e.s.d. in the dihedral angle between two l.s. planes) are estimated using the full covariance matrix. The cell e.s.d.'s are taken into account individually in the estimation of e.s.d.'s in distances, angles and torsion angles; correlations between e.s.d.'s in cell parameters are only used when they are defined by crystal symmetry. An approximate (isotropic) treatment of cell e.s.d.'s is used for estimating e.s.d.'s involving l.s. planes.
Refinement. Refinement of *F*^2^ against ALL reflections. The weighted *R*-factor *wR* and goodness of fit *S* are based on *F*^2^, conventional *R*-factors *R* are based on *F*, with *F* set to zero for negative *F*^2^. The threshold expression of *F*^2^ > σ(*F*^2^) is used only for calculating *R*-factors(gt) *etc*. and is not relevant to the choice of reflections for refinement. *R*-factors based on *F*^2^ are statistically about twice as large as those based on *F*, and *R*-factors based on ALL data will be even larger.

### Fractional atomic coordinates and isotropic or equivalent isotropic displacement parameters (Å^2^)

**Table:**

	*x*	*y*	*z*	*U*_iso_*/*U*_eq_	
N1	0.71730 (16)	0.01395 (14)	0.43192 (9)	0.0305 (3)	
H1	0.6421	−0.0558	0.4543	0.037*	
N2	0.96136 (17)	0.23142 (14)	0.36451 (9)	0.0322 (3)	
H2	1.0403	0.2993	0.3431	0.039*	
N3	0.86203 (18)	0.59454 (17)	−0.10289 (9)	0.0406 (3)	
O1	0.53849 (14)	0.20004 (12)	0.47998 (8)	0.0398 (3)	
O2	0.79757 (16)	0.42195 (12)	0.42271 (8)	0.0412 (3)	
O3	0.54289 (17)	0.39843 (12)	0.20331 (9)	0.0467 (3)	
O4	0.49185 (15)	0.61637 (12)	0.28917 (8)	0.0383 (3)	
H4A	0.4479	0.5555	0.3277	0.058*	
O5	0.85764 (19)	0.45403 (16)	−0.11072 (9)	0.0569 (4)	
O6	0.9246 (2)	0.67836 (17)	−0.16454 (10)	0.0640 (4)	
O7	0.7393 (2)	1.14774 (14)	0.02107 (10)	0.0599 (4)	
O8	0.62819 (19)	1.11218 (13)	0.17111 (9)	0.0538 (3)	
H8	0.6236	1.2062	0.1725	0.081*	
O9	0.32657 (14)	0.43362 (12)	0.40853 (8)	0.0369 (3)	
H9A	0.4013	0.3730	0.4352	0.055*	
H9B	0.3007	0.4804	0.4643	0.055*	
C1	0.67867 (19)	0.16099 (17)	0.44281 (10)	0.0293 (3)	
C2	0.8180 (2)	0.28385 (17)	0.40886 (10)	0.0296 (3)	
C3	0.9929 (2)	0.07624 (17)	0.35027 (10)	0.0299 (3)	
C4	1.1426 (2)	0.0307 (2)	0.30139 (12)	0.0417 (4)	
H4	1.2255	0.1047	0.2770	0.050*	
C5	1.1677 (2)	−0.1238 (2)	0.28923 (13)	0.0481 (4)	
H5	1.2657	−0.1545	0.2547	0.058*	
C6	1.0477 (2)	−0.2347 (2)	0.32807 (12)	0.0436 (4)	
H6	1.0674	−0.3389	0.3208	0.052*	
C7	0.8999 (2)	−0.19105 (18)	0.37722 (11)	0.0346 (3)	
H7	0.8201	−0.2652	0.4037	0.042*	
C8	0.87052 (19)	−0.03525 (16)	0.38704 (10)	0.0280 (3)	
C9	0.63865 (19)	0.64015 (17)	0.13886 (10)	0.0296 (3)	
C10	0.7140 (2)	0.57178 (18)	0.05592 (10)	0.0324 (3)	
H10	0.7161	0.4648	0.0475	0.039*	
C11	0.7857 (2)	0.66751 (18)	−0.01360 (10)	0.0334 (3)	
C12	0.7849 (2)	0.82652 (19)	−0.00467 (11)	0.0366 (4)	
H12	0.8342	0.8876	−0.0530	0.044*	
C13	0.7085 (2)	0.89318 (18)	0.07831 (11)	0.0334 (3)	
C14	0.6363 (2)	0.79974 (17)	0.14970 (10)	0.0318 (3)	
H14	0.5858	0.8448	0.2054	0.038*	
C15	0.5552 (2)	0.53871 (17)	0.21328 (10)	0.0312 (3)	
C16	0.6963 (2)	1.06385 (19)	0.08583 (12)	0.0394 (4)	

### Atomic displacement parameters (Å^2^)

**Table:**

	*U*^11^	*U*^22^	*U*^33^	*U*^12^	*U*^13^	*U*^23^
N1	0.0280 (6)	0.0267 (6)	0.0384 (6)	−0.0004 (5)	0.0113 (5)	0.0073 (5)
N2	0.0322 (7)	0.0281 (7)	0.0385 (6)	−0.0004 (5)	0.0157 (5)	0.0061 (5)
N3	0.0386 (7)	0.0518 (9)	0.0324 (7)	0.0076 (7)	0.0079 (5)	0.0010 (6)
O1	0.0335 (6)	0.0351 (6)	0.0562 (7)	0.0079 (5)	0.0223 (5)	0.0112 (5)
O2	0.0493 (7)	0.0261 (6)	0.0520 (6)	0.0059 (5)	0.0213 (5)	0.0047 (5)
O3	0.0666 (8)	0.0256 (6)	0.0507 (7)	0.0053 (5)	0.0202 (6)	0.0022 (5)
O4	0.0488 (7)	0.0299 (6)	0.0394 (6)	0.0045 (5)	0.0195 (5)	0.0032 (4)
O5	0.0747 (9)	0.0491 (8)	0.0510 (7)	0.0162 (7)	0.0232 (6)	−0.0055 (6)
O6	0.0832 (10)	0.0687 (9)	0.0458 (7)	0.0048 (8)	0.0337 (7)	0.0107 (7)
O7	0.0803 (10)	0.0387 (7)	0.0648 (8)	0.0053 (7)	0.0193 (7)	0.0181 (6)
O8	0.0816 (9)	0.0283 (6)	0.0547 (7)	0.0125 (6)	0.0166 (6)	0.0037 (5)
O9	0.0390 (6)	0.0332 (6)	0.0405 (6)	0.0059 (5)	0.0122 (4)	0.0037 (4)
C1	0.0286 (7)	0.0300 (8)	0.0309 (7)	0.0042 (6)	0.0081 (6)	0.0049 (6)
C2	0.0324 (8)	0.0289 (8)	0.0288 (7)	0.0032 (6)	0.0089 (5)	0.0038 (6)
C3	0.0314 (8)	0.0296 (8)	0.0298 (7)	0.0044 (6)	0.0069 (6)	0.0026 (6)
C4	0.0372 (9)	0.0455 (10)	0.0463 (9)	0.0079 (7)	0.0177 (7)	0.0080 (7)
C5	0.0454 (10)	0.0500 (11)	0.0550 (10)	0.0196 (8)	0.0217 (8)	0.0039 (8)
C6	0.0488 (10)	0.0343 (9)	0.0491 (9)	0.0145 (8)	0.0049 (7)	−0.0012 (7)
C7	0.0359 (8)	0.0295 (8)	0.0381 (7)	0.0023 (6)	0.0026 (6)	0.0038 (6)
C8	0.0278 (7)	0.0294 (8)	0.0270 (6)	0.0033 (6)	0.0038 (5)	0.0015 (6)
C9	0.0293 (7)	0.0292 (8)	0.0304 (7)	0.0040 (6)	0.0036 (5)	0.0005 (6)
C10	0.0327 (8)	0.0307 (8)	0.0338 (7)	0.0047 (6)	0.0040 (6)	0.0001 (6)
C11	0.0315 (8)	0.0390 (9)	0.0299 (7)	0.0049 (7)	0.0055 (6)	−0.0008 (6)
C12	0.0348 (8)	0.0405 (9)	0.0352 (7)	0.0015 (7)	0.0057 (6)	0.0094 (7)
C13	0.0315 (8)	0.0317 (8)	0.0363 (7)	0.0023 (6)	0.0010 (6)	0.0036 (6)
C14	0.0325 (8)	0.0318 (8)	0.0313 (7)	0.0049 (6)	0.0047 (6)	0.0004 (6)
C15	0.0332 (8)	0.0281 (8)	0.0328 (7)	0.0056 (6)	0.0051 (6)	0.0003 (6)
C16	0.0402 (9)	0.0320 (9)	0.0455 (9)	0.0020 (7)	0.0018 (7)	0.0060 (7)

### Geometric parameters (Å, °)

**Table:**

N1—C1	1.3360 (18)	C3—C4	1.391 (2)
N1—C8	1.3972 (17)	C4—C5	1.373 (2)
N1—H1	0.8600	C4—H4	0.9300
N2—C2	1.3428 (17)	C5—C6	1.389 (3)
N2—C3	1.3928 (19)	C5—H5	0.9300
N2—H2	0.8600	C6—C7	1.376 (2)
N3—O5	1.2147 (19)	C6—H6	0.9300
N3—O6	1.2158 (18)	C7—C8	1.390 (2)
N3—C11	1.4764 (18)	C7—H7	0.9300
O1—C1	1.2365 (16)	C9—C14	1.386 (2)
O2—C2	1.2268 (18)	C9—C10	1.3900 (19)
O3—C15	1.2098 (18)	C9—C15	1.492 (2)
O4—C15	1.3114 (16)	C10—C11	1.381 (2)
O4—H4A	0.8200	C10—H10	0.9300
O7—C16	1.201 (2)	C11—C12	1.379 (2)
O8—C16	1.327 (2)	C12—C13	1.388 (2)
O8—H8	0.8200	C12—H12	0.9300
O9—H9A	0.8597	C13—C14	1.388 (2)
O9—H9B	0.8598	C13—C16	1.491 (2)
C1—C2	1.514 (2)	C14—H14	0.9300
C3—C8	1.390 (2)		
			
C1—N1—C8	125.04 (12)	C6—C7—C8	119.52 (15)
C1—N1—H1	117.5	C6—C7—H7	120.2
C8—N1—H1	117.5	C8—C7—H7	120.2
C2—N2—C3	125.43 (12)	C3—C8—C7	120.28 (13)
C2—N2—H2	117.3	C3—C8—N1	118.07 (12)
C3—N2—H2	117.3	C7—C8—N1	121.64 (13)
O5—N3—O6	123.82 (13)	C14—C9—C10	120.07 (13)
O5—N3—C11	118.00 (13)	C14—C9—C15	120.96 (12)
O6—N3—C11	118.17 (14)	C10—C9—C15	118.95 (13)
C15—O4—H4A	109.5	C11—C10—C9	117.97 (14)
C16—O8—H8	109.5	C11—C10—H10	121.0
H9A—O9—H9B	98.3	C9—C10—H10	121.0
O1—C1—N1	123.40 (13)	C12—C11—C10	123.11 (13)
O1—C1—C2	119.59 (13)	C12—C11—N3	118.92 (13)
N1—C1—C2	117.01 (12)	C10—C11—N3	117.95 (14)
O2—C2—N2	123.62 (14)	C11—C12—C13	118.30 (14)
O2—C2—C1	120.46 (12)	C11—C12—H12	120.8
N2—C2—C1	115.92 (12)	C13—C12—H12	120.8
C8—C3—C4	119.61 (14)	C12—C13—C14	119.81 (14)
C8—C3—N2	118.35 (12)	C12—C13—C16	118.98 (14)
C4—C3—N2	122.04 (14)	C14—C13—C16	121.12 (13)
C5—C4—C3	119.81 (16)	C9—C14—C13	120.74 (13)
C5—C4—H4	120.1	C9—C14—H14	119.6
C3—C4—H4	120.1	C13—C14—H14	119.6
C4—C5—C6	120.48 (14)	O3—C15—O4	123.24 (14)
C4—C5—H5	119.8	O3—C15—C9	123.34 (13)
C6—C5—H5	119.8	O4—C15—C9	113.40 (12)
C7—C6—C5	120.25 (15)	O7—C16—O8	123.72 (15)
C7—C6—H6	119.9	O7—C16—C13	123.92 (15)
C5—C6—H6	119.9	O8—C16—C13	112.33 (13)
			
C8—N1—C1—O1	177.60 (13)	C15—C9—C10—C11	178.18 (12)
C8—N1—C1—C2	−3.47 (19)	C9—C10—C11—C12	−0.3 (2)
C3—N2—C2—O2	177.86 (13)	C9—C10—C11—N3	−178.69 (12)
C3—N2—C2—C1	−1.7 (2)	O5—N3—C11—C12	−178.32 (14)
O1—C1—C2—O2	3.7 (2)	O6—N3—C11—C12	1.3 (2)
N1—C1—C2—O2	−175.29 (13)	O5—N3—C11—C10	0.1 (2)
O1—C1—C2—N2	−176.70 (13)	O6—N3—C11—C10	179.69 (14)
N1—C1—C2—N2	4.33 (18)	C10—C11—C12—C13	0.1 (2)
C2—N2—C3—C8	−1.9 (2)	N3—C11—C12—C13	178.43 (13)
C2—N2—C3—C4	178.47 (14)	C11—C12—C13—C14	0.2 (2)
C8—C3—C4—C5	0.4 (2)	C11—C12—C13—C16	−176.43 (13)
N2—C3—C4—C5	180.00 (14)	C10—C9—C14—C13	0.1 (2)
C3—C4—C5—C6	−1.9 (3)	C15—C9—C14—C13	−177.81 (12)
C4—C5—C6—C7	1.4 (3)	C12—C13—C14—C9	−0.3 (2)
C5—C6—C7—C8	0.5 (2)	C16—C13—C14—C9	176.26 (13)
C4—C3—C8—C7	1.5 (2)	C14—C9—C15—O3	174.98 (14)
N2—C3—C8—C7	−178.08 (12)	C10—C9—C15—O3	−2.9 (2)
C4—C3—C8—N1	−177.39 (13)	C14—C9—C15—O4	−3.3 (2)
N2—C3—C8—N1	3.02 (19)	C10—C9—C15—O4	178.79 (12)
C6—C7—C8—C3	−2.0 (2)	C12—C13—C16—O7	4.3 (2)
C6—C7—C8—N1	176.89 (13)	C14—C13—C16—O7	−172.28 (16)
C1—N1—C8—C3	−0.2 (2)	C12—C13—C16—O8	−177.21 (14)
C1—N1—C8—C7	−179.10 (13)	C14—C13—C16—O8	6.2 (2)
C14—C9—C10—C11	0.2 (2)		

### Hydrogen-bond geometry (Å, °)

**Table:**

*D*—H···*A*	*D*—H	H···*A*	*D*···*A*	*D*—H···*A*
N2—H2···O6^i^	0.86	2.40	2.9632 (18)	123.
N2—H2···O9^ii^	0.86	2.33	3.0071 (17)	136.
N1—H1···O1^iii^	0.86	2.02	2.8723 (17)	173.
O9—H9B···O2^iv^	0.86	1.89	2.7456 (15)	171.
O8—H8···O3^v^	0.82	1.86	2.6381 (17)	159.
O9—H9A···O1	0.86	1.97	2.8220 (15)	168.
O4—H4A···O9	0.82	1.78	2.5962 (15)	173.

Symmetry codes: (i) −*x*+2, −*y*+1, −*z*; (ii) *x*+1, *y*, *z*; (iii) −*x*+1, −*y*, −*z*+1; (iv) −*x*+1, −*y*+1, −*z*+1; (v) *x*, *y*+1, *z*.
